# Validation of a digital photographic method for assessment of dietary quality of school lunch sandwiches brought from home

**DOI:** 10.3402/fnr.v57i0.20243

**Published:** 2013-07-12

**Authors:** Marianne S. Sabinsky, Ulla Toft, Klaus K. Andersen, Inge Tetens

**Affiliations:** 1 Division of Nutrition, National Food Institute, Technical University of Denmark, Søborg Denmark; 2 Research Centre for Prevention and Health, Glostrup University Hospital, Glostrup, Denmark; 3 The Danish Cancer Society, Copenhagen, Denmark

**Keywords:** food intake, packed lunches, diet assessment, children

## Abstract

**Background:**

It is a challenge to assess children's dietary intake. The digital photographic method (DPM) may be an objective method that can overcome some of these challenges.

**Objective:**

The aim of this study was to evaluate the validity and reliability of a DPM to assess the quality of dietary intake from school lunch sandwiches brought from home among children aged 7–13 years.

**Design:**

School lunch sandwiches (*n*=191) were prepared to represent randomly selected school lunch sandwiches from a large database. All components were weighed to provide an objective measure of the composition. The lunches were photographed using a standardised DPM. From the digital images, the dietary components were estimated by a trained image analyst using weights or household measures and the dietary quality was assessed using a validated Meal Index of Dietary Quality (Meal IQ). The dietary components and the Meal IQ obtained from the digital images were validated against the objective weighed foods of the school lunch sandwiches. To determine interrater reliability, the digital images were evaluated by a second image analyst.

**Results:**

Correlation coefficients between the DPM and the weighed foods ranged from 0.89 to 0.97. The proportion of meals classified in the same or an adjacent quartile ranged from 98% (starch) to 100% (fruits, vegetables, fish, whole grain, and Meal IQ). There was no statistical difference between fish, fat, starch, whole grains, and Meal IQ using the two methods. Differences were found for fruits and vegetables; Bland–Altman analyses showed a tendency to underestimate high amounts of these variables using the DPM. For interrater reliability, kappa statistics ranged from 0.59 to 0.82 across the dietary components and Meal IQ.

**Conclusions:**

The standardised DPM is a valid and reliable method for assessing the dietary quality of school lunch sandwiches brought from home.

Childhood represents an important life stage for the development of healthy nutritional behaviour, and some evidence exists that nutritional behaviour tracks from childhood into adulthood (1). The dietary habits of children in Denmark (2), as well as for children in other Western countries, call for improvement (3). Assessment of children's dietary intake may be complicated, and inaccurate reporting from both children and parents in dietary surveys has been recognised as a challenge (4). Weighed food records, food diaries, food frequency questionnaires, diet histories, and 24-h dietary recalls are all common methods for estimating dietary intake; however, these methods rely on self-reporting with a relatively high respondent burden.

The accuracy of self-reported methods has been questioned. Studies using doubly labelled water have shown that misreporting of food intake is a common problem for these methods (5–7). Especially when collecting data on dietary intake in a paediatric population, self-reported methods become a challenge. Before the age of 12, children have not yet developed the cognitive skills required by the self-reported methods (4). One of the particular challenges among children is quantification of the dietary intake, and it is difficult for children to estimate portion sizes (4, 8). Thus, there is a need for valid alternative methods to capture actual dietary intake – for example, to evaluate intervention studies aiming to improve the diets of different population groups, especially children. Collecting and analysing dietary intake data from large samples can be time consuming and expensive, but this is important when designing powerful studies. Recently introduced methods applying new technologies have been used that may improve the quality and accuracy of dietary assessment methods (9). These methods may also prove useful for collecting data from a large population.

The digital photographic method (DPM) is a relatively new method. It overcomes children's recall problems and difficulties in estimating portion sizes, and it also minimises the burden of the respondent. The method is unobtrusive, highly reliable, and highly valid when used to estimate the food intake of individual meals of adults and school children in cafeteria settings (10–13). The DPM is also appropriate for collecting data from a larger population group.

It is relatively easy to get information on the composition of lunches provided by the schools, because recipes are available and through them more information on the non-visual food items; furthermore, the meals are often standardised. However, it can be a major challenge to collect objective data on lunches brought from home. In Denmark, school lunches brought from home usually comprise open sandwiches (often on rye bread) with spread and cold sliced meat, sometimes with fruits and vegetables (14). To our knowledge, the method has not yet been tested on this type of meal.

The aim of this study was to evaluate the validity and reliability of a DPM to assess the quality of dietary intake from school lunch sandwiches brought from home among children aged 7–13 years.

## Methods

### Study sample

A total of 191 school lunch sandwiches were prepared based on digital images from a database comprising 2735 school lunch sandwiches brought from home. The database was developed as part of another project where school lunch sandwiches were collected from 8 schools representing different geographical areas in Denmark and from children aged 7–13 years. The size of the study sample was chosen to ensure presence of all relevant food components examined in the dietary assessment procedure (especially fish and snack products) described below. Around 200 lunches would ensure this aspect and because there were 8 schools and two age groups, 12 lunches from each age group and from each of the 8 schools were randomly selected – in total 192 lunches. One meal was excluded because it consisted of only beverages. When the digital images were collected for the database all children were asked to show clearly any non-visible food items (like spreads). During the preparation of the school lunch sandwiches the weight in grams of each food component was registered using a Soehnle 8026 digital balance (0–1,000 g=1 g, 1,000–2,000 g=2 g). A digital image was taken of the final lunch following the procedure described below.

### DPM procedure

**Fig. 1:**
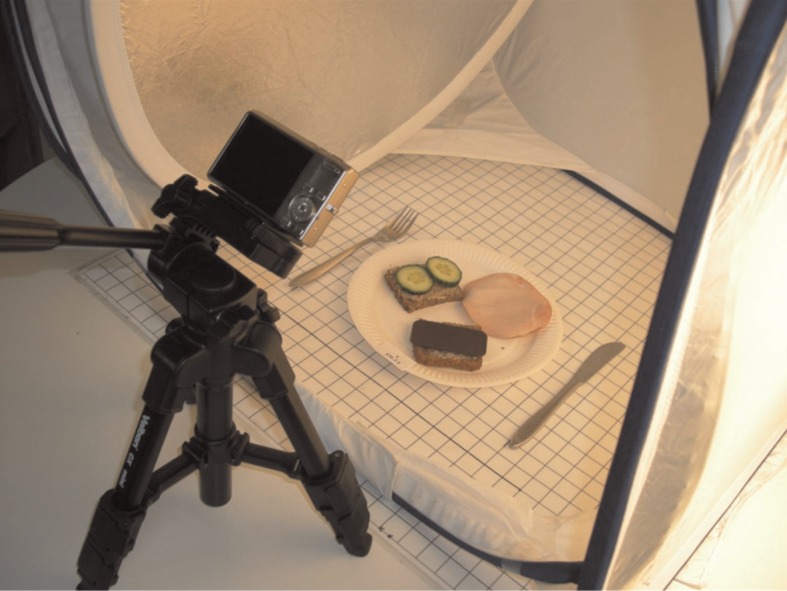
The standardised digital photographic method.

A standardised DPM was developed to collect data on the school lunch sandwiches. The meals were photographed using a digital camera (Nikon S700) mounted on a tripod with the lens 0.37 m above the meal with a camera angle of approximately 45° – a procedure that allows visibility of the foods in three dimensions in a digital image. To standardise the digital images, a placemat (0.6×0.6 m) with markings for placement of the plate and some standardised cutlery were fixed to a table. The placemat was divided into squares of 2×2 cm to support the estimation of the size and weight of the different food items. Markings were also made for where to place the camera tripod. To optimise and standardise the quality of the digital images, a cube light was used (Fig. 1). The research staff attended a training session on the use of the DPM before the data were collected.

### Validation of the DPM

#### The Meal Index of dietary quality

**Fig. 2:**
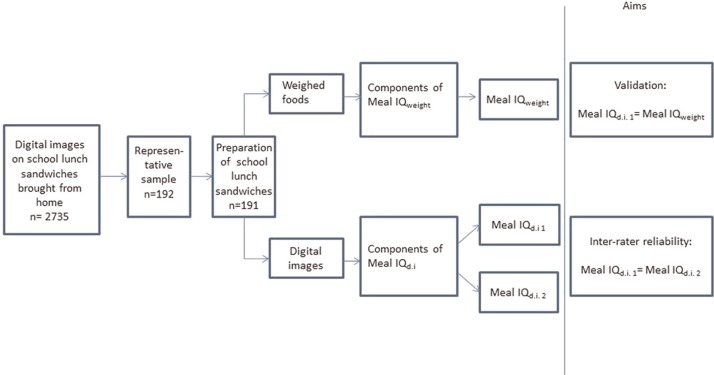
Study design. d.i.: digital images.

A Meal IQ that was developed as a scoring system and published earlier (15) was applied as the tool to assess the dietary quality from the digital images and from the weighed school lunch sandwiches (Fig. 2). The Meal IQ consists of the following seven components based on dietary issues related to children aged 7–13 years and the visibility of the components: total fat, saturated fat, whole grain, snack products, fish, fruits, and vegetables. From these components, a total Meal IQ score is obtained.

Fruits, vegetables, and fish were estimated in grams. To estimate total fat, saturated fat, whole grain, and snack products in the lunch meals, unit sizes were defined in terms of household measures, such as slices, cups, and pieces (16). The total Meal IQ score for a single meal can range from 0 to 28 [for more details on the Meal IQ, see Ref. (15)].

#### Assessment of dietary quality

The components of the Meal IQ and the total Meal IQ score were determined from the objectively weighed 191 school lunch sandwiches. Fruits, vegetables, and fish were already registered in grams, and although weights in grams were assigned to each of the units, it was possible from the registered weights of the food items to calculate the number of fat, saturated fat, starchy, and whole-grain units (to measure the relative (total) fat content of the meal, the number of fat units was subtracted from the number of starchy food units).

The components of the Meal IQ and the total Meal IQ score were also determined from the digital images. To support the conversion of food items in the digital images into weights and unit sizes, reference material was developed. The reference foods were selected to represent the foods most frequently consumed at school lunch by children who brought lunches from home, selected on the basis of the 191 meals representing the study sample. Each food item was photographed in up to eight different portion sizes, and prepared or cut in different ways. The food items were also photographed in different positions on the plate – at the back and the front and at one of the sides of the plate. The reference foods were photographed with exactly the same camera angle and distance from the food, using a cube light so that the apparent size of all foods remained constant across the digital images. These reference foods were supplemented with material from a previous study also using a standardised DPM (17), which were also relevant for the estimation of school lunches. The total collection of photographed reference foods consisted of seven different fruits; 16 vegetables; six fish; nine starchy foods such as bread, rice, pasta, and potatoes; and 22 fatty foods such as butter or spread, meat, and dressing.

Some food items are in standardised portions. These products were not photographed but instead presented in reference lists. Some fatty foods (e.g. sliced meat) were presented in a reference list containing information about typical portion sizes and information on content of fat per 100 g and per portion of the food item, which is necessary for estimating the fat units [see Ref. (15)]. Different fish products were also presented in a reference list with information on the content of fish in a mixed product (e.g. tuna in tuna fish salad spread). Information on starchy food products was also put in a reference list, and in addition to the information on the weight of standard portions, information was also given regarding whether the product was categorised as whole grain. Finally, a reference list of snack products and their content of fat and starch per standard portion was available.

If food items that did not make their fat content visible were presented in the digital images, for example, we used data from GfK (Gesellschaft für Konsumforschung) Denmark, which does market research, to determine the type of product. The assessment was in these cases based on information on the most used product of the category (18). If the digital images showed composite dishes or products for which no declaration was available, for example because the dishes or products were homemade, data from the Danish National Survey of Dietary Habits and Physical Activity (2) were used to assess the dietary composition.

A database was developed using Microsoft Excel for the dietary assessment of the 191 digital images in order to make the necessary notes on the dietary components (grams or units) while watching the digital image.

Ten school lunch sandwiches were used to train the image analysts in portion size estimation on the basis of the photographed reference foods and reference lists. Different persons handled the test and reference methods.

The standardised DPM was validated, testing the agreement of the dietary components included in the Meal IQ and the overall Meal IQ score obtained using the digital images and the weighed foods of the lunches (Fig. 2).

### Reliability testing of the DPM

Interrater reliability testing was conducted on the standardised DPM to assess the ability of the method to yield consistent results for the amount of fruits, vegetables, fish, and fat units (inclusive saturated fat units); the amount of starchy units (inclusive units from whole-grain products); the presence of snack products; as well as the overall dietary quality measured by the Meal IQ score by two raters. The two digital-image analysts’ ratings were compared for each dietary component and the total Meal IQ score for the 191 digital images of the school lunches.

### Statistical analysis

Most of the dietary data were non-normally distributed, both before and after log transformation; therefore, medians and 5th and 95th percentiles are presented. The Wilcoxon signed-rank test was used to analyse the difference in dietary components, and the Meal IQ assessed by the DPM and the food record method.

To validate the DPM, correlation coefficients between the selected dietary components and the Meal IQ estimated from the digital images and from the weighed foods were assessed. As the data on dietary intake were not normally distributed, Spearman's correlation coefficient was used (19). The estimated components and the total score of Meal IQ in quartiles were classified. Gross misclassification was defined as classification in the opposite quartile when observed in the highest or lowest quartile. To evaluate the agreement between the continuous variables (fruits, vegetables, and fish) and the Meal IQ score assessed from the digital images and the weighed foods, Bland–Altman plots were made. The limits of agreement were defined as two times the corrected standard deviations of the differences above and below the mean (20).

To test the interrater reliability of the DPM, a weighted kappa statistic was calculated for each of the dietary components and the Meal IQ. To conduct the kappa statistics on the continuous components and the Meal IQ, the variables were divided into 10 groups according to percentiles.

In the analysis specific for fruits, vegetables, and fish, the meals not containing the respective food item were excluded from the analysis in both the validity and reliability testing.

*P*<0.05 was considered statistically significant. All reported *P* values were based on two-sided hypotheses. Statistical analyses were carried out using the SAS statistical software package (version 9.2, SAS Institute Inc., Cary, NC, USA).

## Results

### Validation of the DPM

Each of the dietary components and the Meal IQ were estimated from the digital images and the weighed foods of the lunches. Table 1 shows the values of the medians and the 5th and 95th percentiles of the dietary components and the total score of the Meal IQ assessed from the two methods. The Wilcoxon signed-rank test showed that no statistical difference was found between fish, fat, starchy, and whole-grain units and the Meal IQ score assessed from the digital images and the weighed foods. The *P* value for the difference between the saturated fat units was also significant (*P*=0.0457). Fruits and vegetables were significantly different when assessed from either the digital images or the weighed foods (Table 1).

**Table 1:** Dietary components and the Meal IQ score estimated from weighed foods and digital images (median and 5th and 95th percentiles)

Components	*n*	Actual content from weighed foods: median (P5, P95)§	Estimated content from digital images: median (P5, P95)§	*P* values for differences	Classified into same/same or adjacent quartile (%)	Correlation coefficients Spearman$
Fruits (g)	67	87 (13; 195)	80 (15; 174)	<0.0001	84/100	0.96
Vegetables (g)	130	52 (10; 141)	48 (10; 125)	0.0003	76/100	0.96
Fish (g)	21	24 (11; 50)	22 (7; 52)	0.0611	81/100	0.89
Fat units	191	1.5 (0; 4.5)	1.5 (0; 5)	0.0855	79/99	0.93
Saturated fat units	191	1.5 (0; 4)	1.5 (0; 4)	0.0457	72/99	0.91
Starchy units	191	1.75 (0.5; 3.5)	1.75 (0.5; 3)	0.2344	74/98	0.89
Whole grain units	191	1 (0; 2.5)	1 (0; 2)	0.0615	87/100	0.96
Meal IQ score	191	16 (5; 20)	16 (6; 20)	0.3394	80/100	0.97

§ P5: 5th percentile; P95: 95th percentile.

$ All Spearman's correlation coefficients were significant, *P*<0.001.

*P* values for Wilcoxon signed-rank test and cross-classification and correlation analysis between values estimated by the digital and the weighed foods.

The Spearman correlation coefficients between the dietary components and the Meal IQ estimated from the digital images or the weighed foods were highest for the Meal IQ score (*r*=0.97) and lowest for fish and starchy units (*r*=0.89) (Table 1).

The proportion of meals classified in the same or adjacent quartiles of dietary intake ranged from 98% (starchy units) to 100% (fruits, vegetables, fish, and whole-grain units, and total score of Meal IQ). Gross misclassification was not found for any of the dietary components or the total Meal IQ score (Table 1).

Snack products were present in only 13 of the 191 lunches, and the assessment of the occurrence was correct in all the cases.

**Fig. 3:**
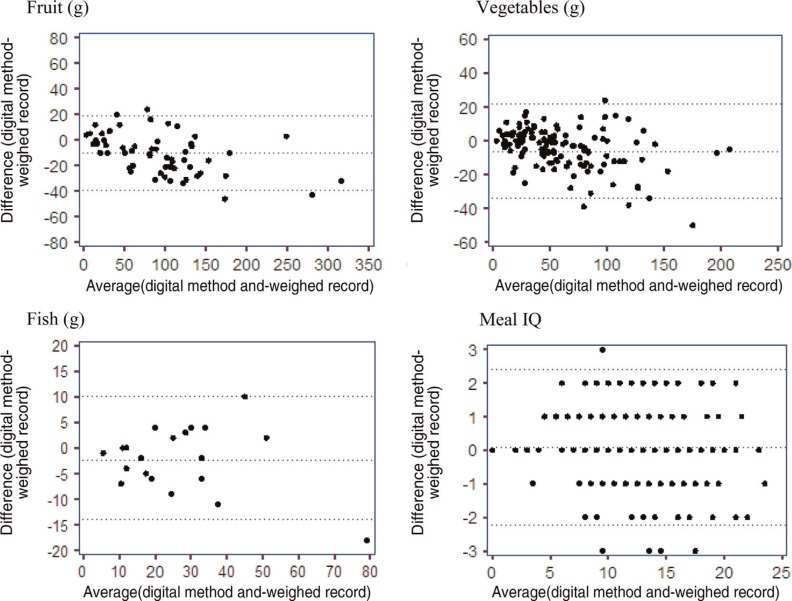
Bland–Altman plots of agreement on the weight of fruits (*n=*67), vegetables (*n=*130), and fish (*n=*21), and the score of the Meal IQ (*n=*191) obtained from the digital photographic method vs. the weighed foods. The *x*-axis shows the mean of the two methods, and the *y*-axis shows the difference between the digital photographic method and the weighed foods. The middle line denotes the mean difference (bias), whereas the top and bottom lines show the upper and lower limits of agreement.

Figure 3 shows the Bland–Altman plots for the continuous dietary variables (fruits, vegetables, and fish) and the Meal IQ score. The amount of fruits estimated from the DPM was compared with the true weight from the weighed food record. The bias was −4.27 g, with the 95% limits of agreement between −29.4 and 20.8 g. Estimation of the amount of vegetables from the DPM had a bias of −6.19 g compared with the weighed food record, and 95% limits of agreement of −34.5 and 22.2 g. When compared with the true amount of fish, the DPM showed a bias of −2.33 g and 95% limits of agreement from −14.7 to 10.0 g. The mean of the difference of the Meal IQ score between the methods was 0.07, and the 95% limits of agreement were ±2.33 around the bias.

### Reliability testing of the DPM

The results for interrater reliability of the dietary components and the Meal IQ are reported in Table 2. Interrater reliability of the estimated dietary components from the DPM showed kappa coefficients that ranged from 0.59 to 0.82 across all components. The variable that yielded the lowest kappa statistic was starchy units. The most reliable variable was the amount of fruits. The Meal IQ yielded a kappa coefficient of 0.76.

**Table 2:** Interrater reliability measures of the digital photographic method using weighted kappa test statistics (*n*=191)

	Interrater	Reliability
Components in Meal IQ	Kappa	95% confidence interval
Fruits	0.82	0.76–0.88
Vegetables	0.79	0.75–0.83
Fish	0.70	0.60–0.79
Fat units	0.69	0.63–0.74
Saturated fat units	0.69	0.64–0.75
Starchy units	0.59	0.52–0.66
Whole-grain units	0.76	0.68–0.84
Presence of snack products	0.80*	0.69–0.91
Meal IQ	0.76	0.72–0.81

* Simple kappa coefficient.

## Discussion

This study is the first to investigate if a standardised DPM is valid and reliable for assessment of selected dietary components and the overall dietary quality of school lunch sandwiches brought from home.

The analysis of the difference between the amount of fruits and vegetables estimated from the digital images shows a difference from the weighed foods, despite almost the same medians and averages of these variables. The Bland–Altman analyses show acceptable limits of agreement for fruits (−29.4 and 20.8 g) and vegetables (−34.5 and 22.2 g), with some variability but on the same level as found by others (17). The smaller sample of the analyses for fruits (*n*=67) and vegetables (*n*=130) affects the variability and the limits of agreement. The Bland–Altman plots illustrate a tendency of increasing underestimation with increasing intake when using the DPM; however, both correlation coefficients were high (*r*=0.96 for both variables), and the cross-classifications illustrate that the ranking of the individual meals was good for both fruits and vegetables (100% was classified in the same or adjacent quartile).

When estimating the defined units of fat, starch, and whole grains from the digital images, no statistical difference from the weighed foods was shown. It is easier to estimate variables in household measures, because they do not require the same degree of accuracy as the variables assessed in grams. But for fish, no difference between the estimated amount from the digital images and the true weight from the food record was found, probably because it is easier to estimate the relatively small amounts of fish compared to the voluminous and especially large quantities of fruits and/or vegetables. The Bland–Altman analysis for fish shows tight limits of agreement (−14.7 to 10.0 g), but also for this food item, the Bland–Altman plots illustrate a tendency towards larger variability of the range of intake. This result must be treated with caution, since the sample for the fish analyses is relatively small (*n*=21). We found a difference in the saturated fat units between the methods, probably because of wrong assessment of the spread used on the bread, since it can be difficult to assess whether it is butter or, for example, margarine.

The Meal IQ consists of both the variables estimated in grams and components assessed in units. Compared to the results from the weighed food record method, the DPM was found to provide a good assessment of the overall dietary quality assessed by the Meal IQ. No difference was found between the Meal IQ score assessed using the two methods (*P*=0.3394). The Bland–Altman plot shows a small bias (0.07), and the limits of agreement are sufficiently tight to suggest good agreement between the methods (−2.26 to 2.40). The Meal IQ is not influenced by the underestimation of fruits and vegetables with increasing intake. Fruits and vegetables are separate components in the Meal IQ, and each component in the Meal IQ scores from 0 to 4 points. If fruits or vegetables are not represented in the meal, the score is 0; and if the meal contains 75 g or more, 4 points are given (15). Further analyses show that the problem of underestimating fruits and vegetables does not exist when estimating weights under 85 g of vegetables and 115 g of fruits.

The correlation coefficients between the dietary components and the Meal IQ assessed from either the DPM or the weighed food record were high (*r*=0.89–0.97). Correlation analyses are often used to validate dietary assessment methods, but correlation coefficients provide only a limited measure of the level of agreement between two methods and should therefore not be used alone. Correlation coefficients depend, for example, on the range of the true quantity in the sample (20). In this study, the correlation coefficients were supplemented with cross-classification of the individual meals. This was also good for the dietary components as well as the Meal IQ. In addition, the Bland–Altman plots used for assessment showed acceptable limits of agreement.

In this study, the interrater reliability was assessed from kappa statistics. The kappa coefficient shows a moderate strength of agreement for the assessment of starchy units by the two raters (*κ*=0.59), very good agreement in estimating the amount of fruits (*κ*=0.82), and good agreement for the other components (*κ*=0.69–0.80) and the Meal IQ (*κ*=0.76) (21). Other studies have evaluated the interrater reliability by calculating intraclass correlation, and they also found a good interrater reliability, with intraclass correlations on the level of 0.80–0.96 for different parameters when using the DPM (12, 13, 17).

The validity and reliability of the method are highly dependent on the skills of the image analysts. To reduce the variability caused by using many raters, intensive training of one or possibly two raters might be preferable to training many raters. Also, future training procedures of image analysts should focus on the underestimation we found, especially for the high amount for fruits and vegetables. Others have also reported underestimation when using the DPM (17, 22, 23).

An advantage of the DPM is the opportunity to collect dietary intake data from large populations (9) (e.g. in intervention studies where dietary data have to be collected and where data on meals should be evaluated). Another advantage is that the burden on the participants is minimal compared to that of other dietary assessment methods, and the method also overcomes the recall problems of children. The visual estimation technique is the most comparable method to the DPM. This method is also shown to be valid and reliable (12) and would overcome some of the same challenges as the DPM. But the advantages of using the DPM instead of the visual estimation technique are rapid collection of the dietary data in the eating environment, convenience for participants and researchers, and the possibility of uninterrupted evaluations of the foods that are studied on the digital images, as opposed to evaluation in the setting for data collection (12).

The most time-consuming step when using the DPM for dietary assessment is the nutritional evaluation, due to reliance on human analysts to estimate food intake and possibly subsequent calculations of the nutrient content. To make the method as cost-effective as possible, we used the Meal IQ in addition to the individual dietary components to assess the dietary quality of the lunches. The Meal IQ score is obtained easily through a simple evaluation process. There is no need to calculate the nutrient content, which would make the calculation of the total score more complex and labour intensive.

It is challenging to assess digital images of school lunch sandwiches brought from home rather than school lunches provided by the school, because recipes and product specifications are not available. But we believe that the method is appropriate for this type of meal as well, because the school lunch sandwiches brought from home normally consist of bread, spread, sliced cold meat, and a piece of fruits or some vegetables, often in relatively standardised portions. A limitation of the DPM may be the dependence of visibility of the food or nutrient of concern. The digital images do not always show details about particular foods (e.g. fat-reduced products). In this study, we used data from GfK Denmark to determine the type of product when the digital image gave too little information (18). In addition, data from the Danish National Survey of Dietary Habits and Physical Activity were used to obtain information on the dietary composition of composite dishes or products. Composite dishes or products are not a big challenge in lunches brought from home for children aged 7–13 years, because they do not often occur. Others have also reported the challenge connected with estimating mixed dishes when assessing dietary intake (12).

The DPM is very unobtrusive and would probably not influence the usual eating patterns of the children, but this is still unclear.

This study shows that the DPM in combination with the Meal IQ is valid and reliable when used to assess the quality of dietary intake from school lunch sandwiches brought from home. There is no reason to believe that the DPM in combination with the Meal IQ would be less accurate with adults. The Meal IQ has to be adjusted just a little, so the cut-off points for the different components included in the Meal IQ are adapted to the official recommendations for adults.

Compared to the more traditional dietary assessment methods, the DPM has mainly been used to collect data on individual meals. Measuring the entire diet of free-living individuals complicates the usability of the DPM. Normally, the respondents are not involved in the collection of data. If the whole diet has to be assessed, it will require that the respondents capture the digital images themselves, thereby introducing greater burden on the respondent and the possibility of increased estimation errors because of lower photo quality and a decreased standardisation of the method. The higher response burden could also affect the compliance negatively. Some studies have examined the possibility of assessing food intake among free-living people. Lassen et al. (17) used a DPM in private homes where the participants were instructed on how to capture digital images on their evening meals to standardise the procedure. Lassen et al. concluded that the DPM for this purpose was valid and feasible. Martin et al. (24) developed a remote food photography method that builds on the DPM. Smartphones were used to capture images of food selection and plate waste and to send the images to a server for food intake estimation. This method was developed specifically to measure energy intake in free-living adults and has proved to be valid.

When food selection and also food intake have to be measured, the standardised DPM is most appropriate when the study population eats in a cafeteria or a classroom, because this makes it possible to collect data on the leftovers. In Denmark, the oldest students often go outside the school during the lunch break, which complicates the use of a standardised DPM. Other methods that incorporate technology would be appropriate for this target group. Boushey et al. (25) found a strong preference for technology methods among adolescents, compared to pen-and-paper records. Maybe using a smartphone as described by Martin et al. (22, 24) would be appropriate to take into account the eating behaviour of young people, or a personal digital assistant with a camera and mobile phone card, as described by Wang et al. (26).

There is much potential in technological methods for assessment of dietary intake, and future advancements are possible (27, 28). Future studies have to examine the possibility of using the DPM to estimate food intake in free-living conditions among children; this aspect would be essential for the possibility to measure the entire diet. Furthermore, research on whether the dietary intake observed during one or more meals is predictive of 24-h dietary intake could also be done.

Automation of the nutrient evaluation could be developed and would improve the cost-effectiveness of the method.

In conclusion, the standardised DPM is a valid and reliable approach for assessing the dietary quality of school lunch sandwiches brought from home for children aged 7–13 years. The method does not rely on the respondents to estimate portion sizes and overcomes the recall problems that exist when collecting dietary data on children. The method is cost-effective and enables data collection for large numbers of people. The method is potentially useful for evaluating the effect of different intervention programmes on dietary behaviours from diverse population groups across different ages.
